# Editorial: Photosynthetic Efficiency Under Multiple Stress Conditions: Prospects for Increasing Crop Yields

**DOI:** 10.3389/fpls.2022.893730

**Published:** 2022-04-28

**Authors:** Fabricio Eulálio Leite Carvalho, Maxwell Adam Ware, Milton Costa Lima Neto, Iker Aranjuelo

**Affiliations:** ^1^La Suiza Research Center, Colombian Agricultural Research Corporation (Agrosavia), Rionegro, Colombia; ^2^Department of Biology, Colorado State University, Fort Collins, CO, United States; ^3^Bioscience Institute, São Paulo State University (UNESP), São Vicente, Brazil; ^4^Consejo Superior de Investigaciones Científicas (CSIC)-Gobierno de Navarra, Sustainable Agriculture and Climate Change Lab Navarra, Agrobiotechnology Institute (IdAB), Navarra, Spain

**Keywords:** photosynthesis, nutritional value, combined stresses, integrative approach, crop productivity, land-use efficiency

Understanding the impact of combined stresses on photosynthesis, as occurs in nature, is likely a crucial strategy to increase crop productivity sustainably. Water and salt stresses, excess light, changes in temperature and nutritional requirements, and the co-occurrence of biotic stresses are integrated factors that are translated into complex responses adopted by plants. The subsequent adjustments are generally aimed at the efficient allocation of energy resources, plant survival, and offspring creation. In this aspect, plants have co-existed with these natural phenomena for millions of years. Their immense phenotypic plasticity guarantees effective adaptation and survival to a wide range of environmental variations, including the projected global climate changes. We humans, on the other hand, relying on few crops as our main food source, find ourselves in a more precarious situation in this chain of events. Indeed, we are already threatened by increasingly extreme weather events observed in the present. Starting from the premise that early knowledge is the only way to assure global food security in the short, medium, and long term, we set out to ask the following: what do we actually know about the photosynthetic mechanisms of plants in a systemic context (Lima Neto et al., 2021) that allows us to increase the crop yield, nutritional value, and/or land-use efficiency under field conditions?

Therefore, this was more than a plan to gather knowledge about this important issue within plant science: it was an invitation to launch a challenge to fellow researchers engaged in related projects. We were both surprised and gratified by the number of results and promising conclusions that we were able to gather in this special edition. Among the interesting review papers that we edited here, Ma et al. carried out a conceptual and functional review of the controversial NADPH dehydrogenase complex and its possible functions in regulating cyclic electron transport (CET). Interestingly, Ma et al. highlighted how much of this mechanism is still unknown, notably pointing out the need for more extensive knowledge of the mechanisms of transport and regulation of NDH-CET, especially under abiotic stress conditions. In fact, the cyclic flow of electrons in photosynthesis remains one of the most exciting and enigmatic subjects (Cerqueira et al., 2019). Above all, understanding how these processes can concretely contribute to photoprotection and energy balance in plants subjected to potentially stressful conditions can shed light on future technologies for the improvement of crop resistance.

A great difficulty in transposing interesting results observed under laboratory conditions to the field lies in the fact that in natural environments, plants face a complex network of biotic and abiotic stimuli with multiple integrative potentials associated with non-linear counter-responses in plants. In this context, Chávez-Arias et al. reviewed recent information concerning the physiological, biochemical, and molecular responses of plants to the combination of drought, heat stress, and the biotic stress associated with predatory insects of interest in the maize crop. Interestingly, although most studies approach biotic and abiotic stresses separately, in nature, their combination should be seen more as a rule than an exception, with interactive effects both positive and negative (Varela et al., 2018). In their review, Chávez-Arias et al. highlighted that the combination of some abiotic stresses and arthropod herbivory in maize might increase the production of volatile and non-volatile compounds, resulting in improved response to pest infestations.

One of the most fundamental resources to affect photosynthetic activity is the luminosity factor. Gao et al. reported that changes in light quality, especially regarding the composition of blue and white spectra, can increase defense mechanisms and photosynthetic performance in the green onion, which may elucidate effective strategies to increase the productivity of this transient plant under controlled environments. Working with rice, Xiong et al. evidenced that genotypes with smaller but highly dense stomata may present faster stomatal kinetics associated with higher biomass accumulation under fluctuating light. Stomatal kinetics is a significant feature in plants that could be better explored by breeders in the future, especially in relation to better use of the water resource in conditions of climate change. Additionally, the use of microalgae to design biotechnological strategies and obtain bioproducts of high economic value has been considered a very promising field for applying knowledge about photosynthetic regulation. In this context, Chouhan et al. exploited fatty acids accumulation in two mutants, *pgrl1* and *pgr5*, of *C. reinhardtii* under high light conditions. The technological strategy, in this case, was based on the malfunction of CET and the generation of ROS as a side effect of excess light, which contributed to the improvements in fatty acid yields. Equally important, the effects of low light were also in evidence in this special edition. Gong et al. investigated the expression of genes induced by low light intensity and found that plant hormone signal transduction genes displayed an important role in the shadow acclimation response of mung beans. Also investigating shade responses in strawberries, Choi reported non-photochemical quenching (NPQ), photochemical quenching (qP), and fluorescence decline ratio under a given light condition (RFd) as important markers to test stress states of horticultural crops under a low light intensity condition, which suggest the importance of improved management strategies, such as the use of a supplemental light source to rally crop production in greenhouse conditions.

Drought stress is another important environmental condition that may impact crop yields, especially under climate change scenarios, where rainy and dry seasons became more extreme events. In the present Research Topic, Li et al. reported that WUE may be improved in tomato plants under mild and moderated drought due to ABA effects on stomatal and mesophyll conductance. Indeed, plants trigger several important protective mechanisms to cope with drought stress. Among these mechanisms, the C3-CAM shift is an exciting physiological phenomenon observed in some Bromeliaceous plants (and others). Here, Gonçalves and Mercier shed light on this process by investigating *Guzmania monostachia* under drought stress conditions and found that drought had a positive effect on the C3-CAM shift in the apical leaf part. Urea can positively modulate the CAM-idling photosynthesis, demonstrating an interesting integration between C and N metabolisms in this species. In addition, in the current special topic, salt stress, which also comprises an important osmotic stress component, was investigated by Zong et al. The authors reported a possible halophyte medicinal plant species, *Xanthoceras sorbifolium* Bunge, which exhibited optimal growth under 140 mM NaCl. The use of extremophile plants provides alternative cash crops to producers living in regions affected by unfavorable environmental conditions may also represent an essential strategy in the future.

High temperature and high levels of CO_2_, projected to be important abiotic conditions during global warming, were probed by several authors in the Research Topic. In this context, Suárez et al. performed a 30-year study regarding heat-resistant beans growing in the western Amazon region. The authors revealed the importance of changes in night temperature for bean productivity in this region and used several photosynthetic parameters as traits to identify the most water-efficient and heat-resistant genotypes. Another important study under field conditions was carried out by Zhou et al., evaluating simulated conditions of environmental warming in alpine plant communities of the Tibetan plateau. The authors reported that under climate warming, the photosynthetic performance and productivity of meadow communities exhibited significant increases, suggesting changes in future net plant C uptake under climate change scenarios.

Also highlighted in this Research Topic is the potential of biotechnological approaches for the optimized management of crops under high temperatures. Accordingly, Pantoja-Benavides et al. revealed the potential use of phytohormones, especially cytokinins and brassinosteroids, to mitigate heat stress in rice. In parallel, Shao et al. reported that the improved availability of magnesium may positively affect the Rubisco activation state in wheat, enhancing photosynthetic activity whilst simultaneously mitigating heat stress. These studies reveal the need for new low-cost management approaches to cope with abiotic stress in crops. Also working with wheat, Li et al. observed that elevated CO_2_ concentrations result in a high ear C sink strength, favoring photochemical quenching in the flag leaves. In rice, Miao et al. reported positive effects on photosynthesis induced by high CO_2_ concentrations, especially in the jointing stage. These authors also reported the need for better adjustment in parameters employed for estimating stomatal conductance models, which must be adjusted according to genotype and CO_2_ concentrations. Remarkably, all these studies concerning high CO_2_ and temperature simulations revealed the great complexity in understanding present and future global climate change scenarios and especially the need to investigate the combined occurrence of both phenomena deeper.

Wang et al. evaluated the photochemical responses of soybean plants exposed to combined heat and high light stresses. These authors discovered that for soybeans, the combination of stresses was potentially harmful to photosystem II activity. Interestingly, some crop species present a stable or even better response under a combination of stresses, such as cashew plants (Ferreira-Silva et al., 2011; Lima et al., 2018). This reinforces the need to investigate alternative crops to guarantee food security in more affected regions under a global climate-changing scenario. Working with maize, Correia et al. found that lower transpiration associated with high temperatures and vapor pressure deficit promoted a better physiological status in plants exposed to high temperature and/or extended drought. Equally important, studies considering the combination of biotic and abiotic stresses need to be explored in the main species of commercially important plants. In this Research Topic, Mendoza-Vargas et al. reported that drought promoted the early appearance of symptoms and negatively impacted the cape gooseberry plants exposed to fusaric acid or directly inoculated with the fungus *Fusarium oxysporum* f. sp. Physalis (Foph). Therefore, the interaction between stresses can generate positive cross-tolerance effects and trigger additive susceptibility responses, in both cases with great repercussions on plant physiology. Thus, to optimize productivity in these species in the future, integrative investigations of combined stress effects are fundamental.

Finally, this special edition also brought together interesting studies that reveal the importance of understanding photosynthetic mechanisms in the agronomic management of crops. Investigating the potential benefits of biochar use in peanut production, Wang et al. found that biochar application impacted positively the photosynthetic capacity and yields in peanuts, which may have been associated with a better adjustment of carbon and nitrogen metabolism induced using the compound. Working with *Malus domestica*, a fruit tree from temperate zones, Shah et al. raised the hypothesis that the exogenous application of salicylic acid, an important signaling molecule in plants, has the potential to affect chlorophyll synthesis and foliar photosynthetic metabolism positively, thus providing a more favorable environment for the emission of flowering signals originating from the leaves. Although further studies are still needed in this regard, these data illustrate the great complexity of understanding photosynthesis from a systemic perspective to impact crop productivity. In addition, Penzel et al., studying this same fruit species, reinforced the hypothesis that the capacity of light interception is directly associated with fruit mass and soluble solids content. Interestingly, these authors proposed using the leaf area parameter, which is proportional to the amount of light interception capacity, as an early marker for estimating plant productivity in this temperate plant species. Future studies would be very interesting to exploit this relationship in plants from an equatorial environment, where light incidence is commonly excessive.

We believe that the 136 authors who contributed to this Research Topic met the challenge posed in this special edition. Above all, the importance of integrative studies in photosynthesis and the great potential of this knowledge to generate impacts on crop productivity is more than evident here. Fostering this understanding is, therefore, imperative for food security, especially in the face of the great challenge of the 21st century, which focuses on the mitigation of the adverse effects of global warming. We are also grateful for the contribution of all reviewers who added their knowledge and efforts to the realization of this Research Topic. Hopefully, this Research Topic will serve as a basis for encouraging more researchers to take an integrative view of photosynthesis in the context of plant biology and food security.

## Author Contributions

All authors listed have made a substantial, direct, and intellectual contribution to the work and approved it for publication.

## Conflict of Interest

The authors declare that the research was conducted in the absence of any commercial or financial relationships that could be construed as a potential conflict of interest.

## Publisher's Note

All claims expressed in this article are solely those of the authors and do not necessarily represent those of their affiliated organizations, or those of the publisher, the editors and the reviewers. Any product that may be evaluated in this article, or claim that may be made by its manufacturer, is not guaranteed or endorsed by the publisher.
